# Effect of Subglottic Stenosis on Expiratory Sound Using Direct Noise Calculation

**DOI:** 10.3390/app132413197

**Published:** 2023-12-12

**Authors:** Biao Geng, Qian Xue, Scott Thomson, Xudong Zheng

**Affiliations:** 1 Department of Mechanical Engineering, Rochester Institute of Technology, Rochester, NY 14623, USA; 2 Department of Mechanical and Civil Engineering, Brigham Young University-Idaho, Rexburg, ID 83460, USA

**Keywords:** flow-sustained tones, hole tone, whistle, respiratory sound, subglottic stenosis

## Abstract

Subglottic stenosis (SGS) is a rare yet potentially life-threatening condition that requires prompt identification and treatment. One of the primary symptoms of SGS is a respiratory sound that is tonal. To better understand the effect of SGS on expiratory sound, we used direct noise calculation to simulate sound production in a simplified axisymmetric configuration that included the trachea, the vocal folds, the supraglottal tract, and an open environmental space. This study focused on flow-sustained tones and explored the impact of various parameters, such as the SGS severity, the SGS distance, the flowrate, and the glottal opening size. It was found that the sound pressure level (SPL) of the expiratory sound increased with flowrate. SGS had little effect on the sound until its severity approached 75% and SPL increased rapidly as the severity approached 100%. The results also revealed that the tonal components of the sound predominantly came from hole tones and tract harmonics and their coupling. The spectra of the sound were greatly influenced by constricting the glottis, which suggests that respiratory tasks that involve maneuvers to change the glottal opening size could be useful in gathering more information on respiratory sound to aid in the diagnosis of subglottic stenosis.

## 1. Introduction

### 1.1. Subglottic Stenosis and Respiratory Sounds

Subglottic stenosis (SGS) refers to a pathological narrowing of the trachea near the larynx. SGS can be of idiopathic, congenital, or acquired origin (e.g., as a result of prolonged intubation) [1]. Apart from common symptoms like hoarse voice, shortness of breath, and dysphonia, SGS can also lead to adventitious tonal sound during respiration, which is sometimes referred to as stridor [1–4]. For example, P. H. Holinger et al. [4] reported that in infants and children with congenital SGS, 43% presented stridor. On the other hand, stridor is only one of the many possible types of adventitious respiratory sounds, and it arises from other airway obstructions more regularly than from SGS (see, e.g., references [5,6]). Due to the subjective nature of human perception [7] and the variability in the characteristics of stridor and other respiratory sounds, patients with SGS and stridor are sometimes misdiagnosed with other conditions like asthma and pneumonia. Such a misdiagnosis can be life-threatening [1,8,9].

Respiratory sounds are recognized as an integral part in the evaluation of patients with complaints related to the respiratory system and may provide valuable clues about their pathological conditions [10]. Establishing the relationship between the sound, the severity, and the location of the airway obstruction could reduce the use of invasive procedures for diagnosis. Therefore, it is important to both quantify the characteristics of the sounds and to understand the underlying sound generation mechanisms. Research efforts have been made to differentiate respiratory sounds due to upper airway obstruction from other sources, to quantify the acoustic characteristics of the respiratory sounds of obstructed airways, and to study the tonal sound generation by airflow in an obstructed bronchial airway. Baughman & Loudon [11] compared the sound signals of patients with stridor and asthma and found that while the spectrum content was similar, the signal was more intense over the neck than the chest. In patients with stridor, musical sounds occurred during inspiration, while in patients with asthma, they mostly occurred during expiration. Coleman & Schechter [12] used a model consisting of an orifice plate in a variable length tube to study the acoustic effects of airway obstruction. They showed that the noise level increased with stenosis severity and flowrate and suggested that the spacing of peaks in the sound spectrum indicated the location of the stenosis. Basovsky et al. [13] hypothesized and then experimentally showed that tonal sound is produced by nozzle–jet–obstacle structures in obstructed bronchial airways, explained by the hole tone mechanism [14]. Most recently, there are research efforts using the machine learning approach to address the challenges in identifying airway obstructions based on respiratory sounds [15,16].

A few recent studies on SGS have focused on its influence on voice production [17–19]. A common finding was that SGS had no significant effect on the glottal flow until high severity (where the area blockage was 80%~90%). In their experimental study, Hilton & Thomson [18] observed that high-frequency noise became significant by 80% obstruction. However, these studies were performed under the phonation condition where the glottis was closed and the vocal folds vibrated during interaction with the air flow. Thus, the dominant sound source was the pulsatile flow, which was regulated by the vocal fold vibration. This is essentially different from the respiratory condition where the vocal folds are abducted and do not vibrate. Studies that specifically focus on the respiratory sound due to SGS are very limited. van der Velden et al. [20] used numerical simulation to study the sound generated by SGS in a patient-derived realistic geometric airway model, finding that the stridor generated by SGS produced a 15–20 dB higher broadband turbulent noise at higher frequencies compared to the healthy model. Yet, stridor is a tonal or musical sound that sometimes can also be described as “whistling” [5,21–23], which suggests the presence of flow-sustained tones in the airway system containing the SGS.

Flow-sustained tones are a ubiquitous and widely studied phenomenon. The following section reviews four common mechanisms responsible for their occurrence in the context of obstructed airways. This provides a brief physics background to facilitate better understanding of the generation of tonal respiratory sounds and the results presented in this paper.

### 1.2. Mechanisms of Flow-Sustained Tones

Flow-sustained tones involve unstable flows and some form of an amplification–feedback loop. Chanaud [24] categorized aerodynamic whistles into three classes based on the types of feedback, whose occurrence highly depends on the geometric configuration. A survey of previous studies (with the relevant references given below) on flow-sustained tones in various geometric configurations suggest that jet tone, jet–obstacle interaction, jet–obstacle–resonator interaction, and pipe tone could potentially play a role in tonal SGS sound generation. To facilitate further discussion in the paper, each tone mechanism is briefly introduced here.

1. Jet tone. Circular and planar jets exhibit inherent instability due to the shear layer, causing the axial flow speed to oscillate. The Strouhal number of such oscillations is roughly constant, depending on the orifice geometry, such as nozzles or sharp edges [25–27]. For axisymmetric ducted jets, the Strouhal number is approximately 0.5 [28,29]. Small acoustic perturbations can be amplified by the instability of circular jets, leading to shedding vortices in the free boundary layer [29]. Such amplification is selective with the preferred Strouhal number between 0.2 and 1.0 [30,31].
2. Jet–obstacle interaction. Jet–obstacle systems consist of an upstream narrowing, such as through an orifice or a slit, that produces a jet, and an obstacle that is closely located downstream. The tones produced in these systems are often named after the shape of the obstacle, with the most common being the edge tone and the hole tone, both of which tend to operate within a relatively low (<3000) Reynolds number range [31]. In such systems, flow-sustained tones can be generated in a series of vortex modes characterized by the number of vortices between the orifice and the obstacle [32]. While distinct vortex shedding is often observed, it is not necessary for the vortex to impinge on the hole plate to generate the tone [14,33]. Thus, it is more appropriate to regard the vortex as a sign of disturbance than the source of the disturbance in the orifice. As this disturbance is convected downstream, leading to an impingement on the obstacle, it causes a pressure pulse that propagates upstream at sound speed, which is amplified by the jet to form a loop [14,31]. The SGS and the vocal folds form a potential jet–obstacle system.
3. Jet–obstacle with resonator. The addition of a resonator to the jet–obstacle system causes the frequency of the excited tones to approach the resonance frequencies of the resonator, resulting from the coupling of the vortex mode of the jet–obstacle system and the acoustic mode of the resonator [34–36]. Resonance with the resonator also allows the jet–obstacle system to operate in a much larger range of Reynolds numbers (up to 200,000 [31]). Wind instruments, such as recorders and ocarinas, are examples of this type of system. An axisymmetric jet-driven Helmholtz resonator (JDHR) [31], which is essentially a tubular cavity enclosed by two concentric orifice plates, is a special configuration in this category. Although higher modes can exist in the axisymmetric JDHR at high Reynolds numbers [37], previous experiments have mainly focused on exciting the fundamental acoustic mode [31,33,38] for which the acoustic pressure inside the cavity oscillates uniformly. The cavity enclosed by the SGS and the vocal folds forms a potential Helmholtz resonator.
4. Pipe tone. In pipe tone systems, a jet (which is typically circular) is coupled with a pipe resonator. This system differs from the jet–obstacle system in that the acoustic pressure is generated directly at the first and only orifice [39] without any interaction with an obstacle.

SGS in the central airway presents interesting potentials for flow-sustained tones. The narrowing due to the SGS produces a jet flow that is inherently unstable. The vocal folds could function as obstacles, causing acoustic perturbations to the jet flow. The vocal tract could introduce acoustic feedback and form resonance at its formant frequencies. Moreover, the system’s characteristics change with the respiratory effort, the respiratory phase, and the severity and location of the SGS. However, the specific mechanisms and conditions responsible for generating flow-sustained tones in the airway system containing the SGS are not well understood, particularly in relation to the production of stridor.

In this study, we aim to investigate sound production in the human upper airway with SGS using numerical simulation, with a particular focus on flow-sustained tones. The rest of the paper is organized as follows: Section 2 provides an introduction to the simulation setup and its validation. Section 3 presents the results of the parametric study. Section 4 summarizes the mechanisms of tonal sound due to the SGS and discusses this study’s limitations and clinical implications.

## 2. Methods

### 2.1. Simulation Setup

The simulations were conducted using an ANSYS Fluent (R19.2.0, Ansys, Inc., Canonsburg, PA, USA), in which both flow and acoustics were solved simultaneously using the compressible Navier–Stokes equations, which are a so-called direct noise calculation method [40]. While the airflow in the upper airway is of low Mach numbers and can be considered as incompressible, a compressible solver, while computationally expensive, was necessary to capture the coupling between the flow and the sound [41]. The laminar viscous model was used with viscous heating enabled, which meant that the flow was solved using direct numerical simulation (DNS) without any turbulence modeling. This choice was based on the axisymmetric configuration that will be introduced next. The pressure-based solver was used with the Coupled algorithm for pressure-velocity coupling. Air was modeled as an ideal gas with default constant properties (a viscosity of 1.7894 × 10^−5^ kg/(m s), a thermal conductivity of 0.0242 W/(m K), specific heat of 1006.43 J/(kg K), and a molecular weight of 28.966 kg/kmol). For spatial derivatives, pressure was discretized using the second-order scheme and other variables using the QUICK scheme. The second-order implicit time marching scheme was used with a constant time increment size of 5 × 10^−6^ s. A steady flow field was obtained before marching the solution in time.

**Figure 1.**
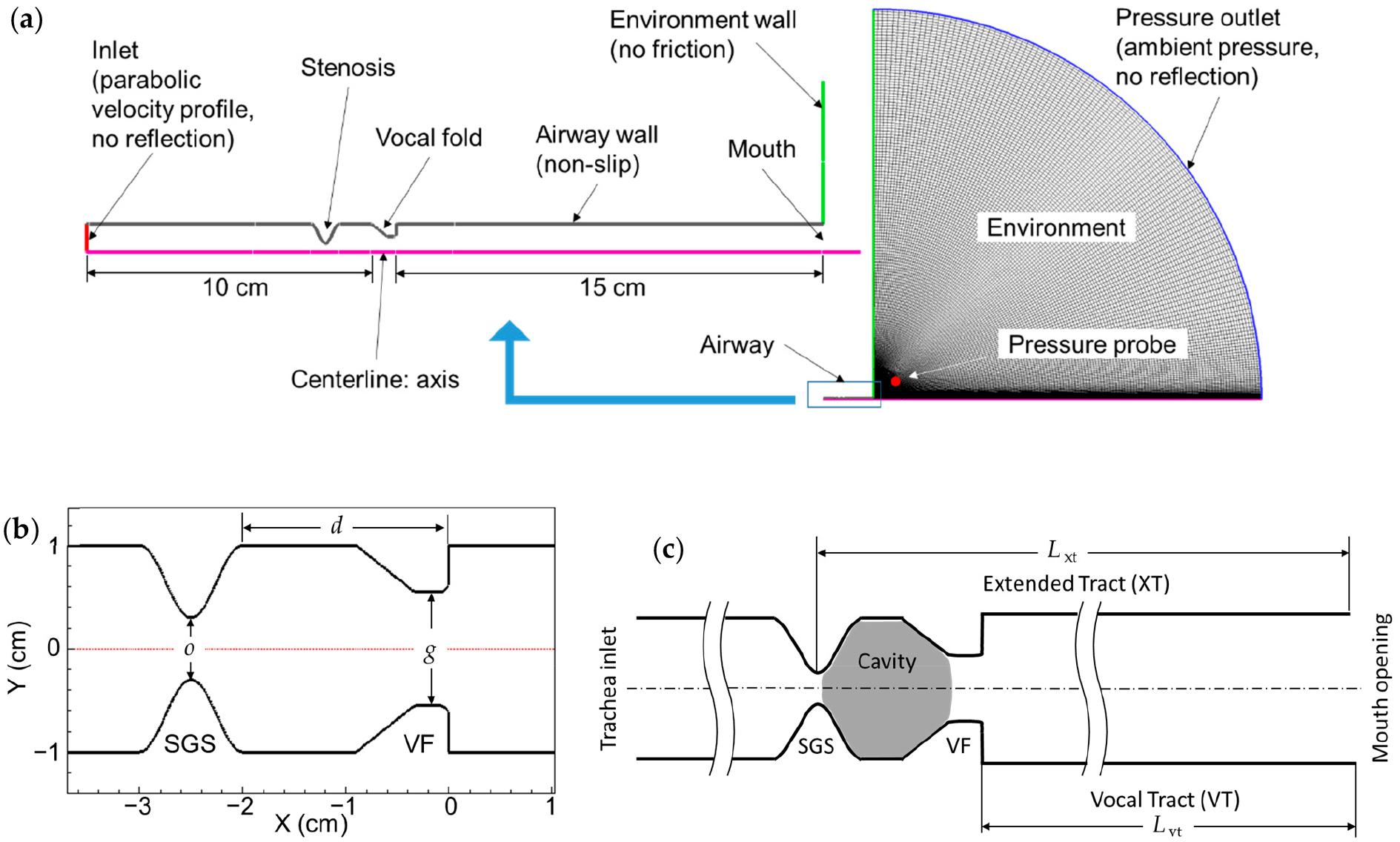
(**a**) Computational domain (to the right) and a zoomed-in view of the tract system showing the setup of boundary conditions. (**b**) SGS–glottis subsystem. In the figure, *o* = 0.6 cm, *g* = 1.1 cm, and *d* = 2.0 cm. (**c**) Sources of acoustic resonance in the system, including the vocal tract (VT, from glottal exit to mouth), the extended tract (XT, from SGS opening to mouth), and the SGS–VFs cavity.

To reduce computational cost and to make the parametric simulations feasible, the simulation domain (and the flow) was assumed to be axisymmetric. In this way, only a two-dimensional (2D) profile section (Figure 1a) needed to be resolved with mesh. The axisymmetric setup enforced the spatial coherence of the vortical structures. It also precluded vortex stretching and, consequently, the production of turbulence. While the glottal jet is more of a 2D structure, axisymmetric configurations have been used in some previous studies on glottal flow and aeroacoustics in phonation [42,43]. It was observed in these studies that, due to lack of vortex stretching and small-scale turbulence, all vortices generated at the glottis survived and convected downstream instead of being dissipated. Axisymmetric configurations have also been used to represent obstructed upper airways [12,44]. Compared to a 2D configuration, the axisymmetric configuration ensured the actual area ratio between stenosis and the vocal tract, which was important in this current study. The effects of the axisymmetric assumption in relation to the present results are discussed in Section 4.3.

The simulation domain (Figure 1) comprises the trachea, true vocal folds, vocal tract, and a half-circular environmental domain. The trachea and vocal tract were simplified as straight tubes with a diameter of 2 cm, which is approximately the average measurement of adult tracheas [45,46]. The length of the trachea and the vocal tract were 10 cm and 15 cm, respectively, based on average adult measurements [46,47]. The radius of the environmental domain was 2 m. A pressure probe was placed 0.1 m from the mouth at a 45-degree angle to the jet (Figure 1a). This was to mimic the typical way that sound is recorded on a human subject using a microphone. Analysis in this paper was based on signals collected using this probe. The solver and domain setup parameters are summarized in Table 1.

The vocal fold profile was defined using the M5 model [48]. The base thickness of the vocal fold (thickness of the lateral surface) was fixed at 0.9 cm. The geometry of the SGS was based on a previous study [17] and was defined by a sinusoidal function of one wavelength. The vertical (inferior–superior) thickness of the SGS at the base remained constant at 1 cm, while the severity of the SGS was adjusted by modifying the amplitude of the sinusoidal function. The location of the SGS was determined by the distance between the superior surface of the vocal fold and the cranial SGS–trachea transition point (as shown in Figure 1b). Three key geometric parameters (*o*, *g*, and *d*) were used to characterize the SGS–glottis subsystem and to denote different parametric cases: *g* represented the glottal opening size; *d* represented the location of the SGS; and *o* represented the minimum diameter of the trachea due to the SGS restriction.

The centerline was designated as the axisymmetric boundary, and the environmental outlet was set as a pressure outlet with a gauge pressure of zero and no acoustic reflection. A parabolic velocity profile was specified at the tracheal inlet, which is a common approach since the flowrate is often reported in the literature. The tracheal inlet was set to have no acoustic reflection to simplify the system, and the solver automatically addressed the reflection at the open mouth. The absorption of the acoustic waves at the inlet and outlet (i.e., no reflection) was realized by using the general non-reflection boundary conditions (NRBCs) in ANSYS Fluent. In this case, the Euler equations were solved in an orthogonal local coordinate system on the boundary to determine the amplitudes of the acoustic waves and the local pressure. This treatment was in effect equivalent to the perfectly matched layer (PML, see e.g., [49]) for acoustic wave absorption. With outgoing waves fully absorbed at the outlet, a much smaller environmental domain could potentially be used. In this study, a large environmental domain was used, so that the ambient pressure could be applied at the boundary with minimum impact on the jet flow.

### 2.2. Solution Independence

**Figure 2.**
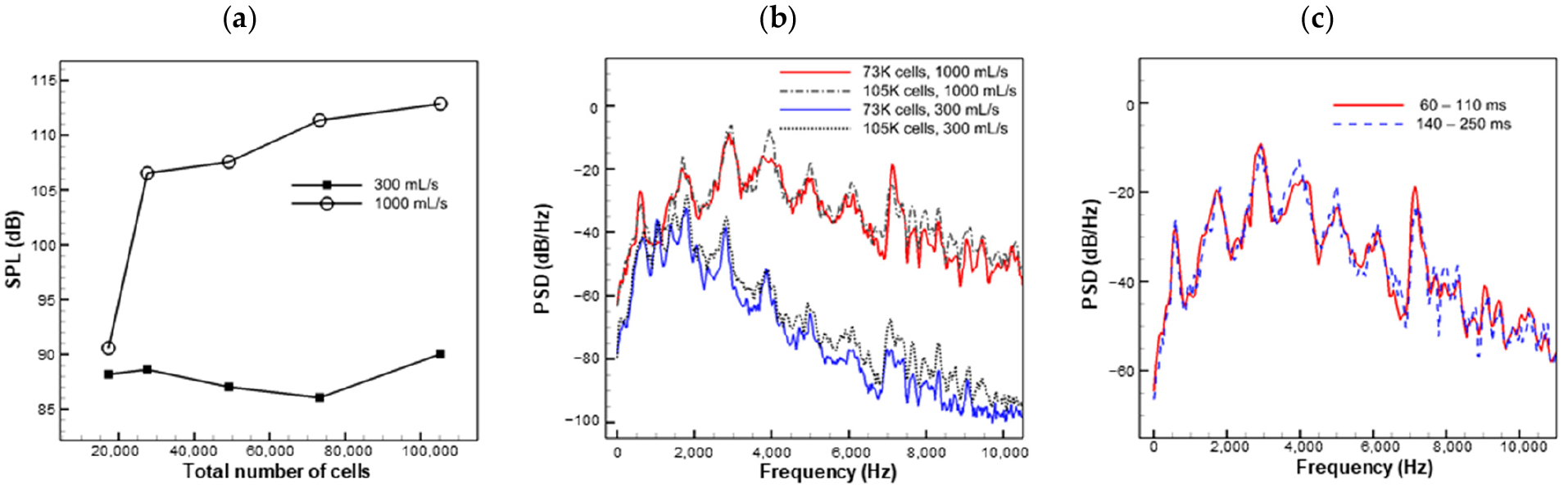
Solution independence. (**a**) Overall sound pressure level versus the number of grid cells. (**b**) Power spectrum of the probed acoustic pressure showing grid independence. (**c**) An example of statistically steady solution.

To ensure grid independence, a multi-step mesh refinement approach was employed. Initially, a coarse non-uniform unstructured mesh was designed to resolve the basic flow features, and, subsequently, the mesh was refined by decreasing the grid interval size by 25% for each successive mesh. Mesh independence was checked for a geometric configuration (*o* = 0.4 cm, *d* = 3.0 cm, and *g* = 0.4 cm) that exhibited the highest sound pressure level in the preliminary simulation results. A total of five meshes were tested at flowrates of 300 mL/s and 1000 mL/s, with the total number of cells ranging from 17 K to 105 K. Figure 2a shows the overall sound pressure level (SPL, see definition in Section 3) versus the number of grid cells. Figure 2b shows a comparison of the acoustic spectra for both flowrates between the two densest meshes. Since the focus of this study was the tonal sound, quantitative comparison was made for the SPL and the primary frequency. The data are summarized in Table 2, with errors in the primary frequency being below 2% and errors in the SPL below 4 dB. The results indicate that the second densest mesh with 73 K cells can predict the location and amplitude of the peaks in the spectrum with adequate accuracy compared to the densest mesh. The minimum grid size for the 73 K mesh was 0.12 mm. The grid was not further refined due to concerns regarding computational speed. It will be shown in the validation case that such grid size can accurately reproduce the experimental results. Moreover, the smaller mesh was favored to considerably reduce the computational cost as the wall time scaled linearly with grid size. The simulation was run using 6 cores on a 3.4 GHz Intel Xeon (Skylake) CPU. For the adopted grid size (73 K cells), each time step took about 3.8 s to converge, and it took about 24 h to run the simulation to 110 ms.

To ensure that the simulation produced statistically steady results, the spectra of the 1000 mL/s case were computed using two different time durations: 60–110 ms and 140–250 ms. As shown in Figure 2c, the two spectra were found to be nearly identical, indicating that the simulation reached statistical steadiness at up to 110 ms. Therefore, most of the cases in the parametric space discussed below were simulated for 110 ms, and an interval of 60–110 ms was used for analysis. However, in cases where the flowrate was very low, resulting in a low sound frequency, the simulation was extended to 220 ms to provide sufficient data for Fourier analysis.

### 2.3. Validation

**Figure 3.**
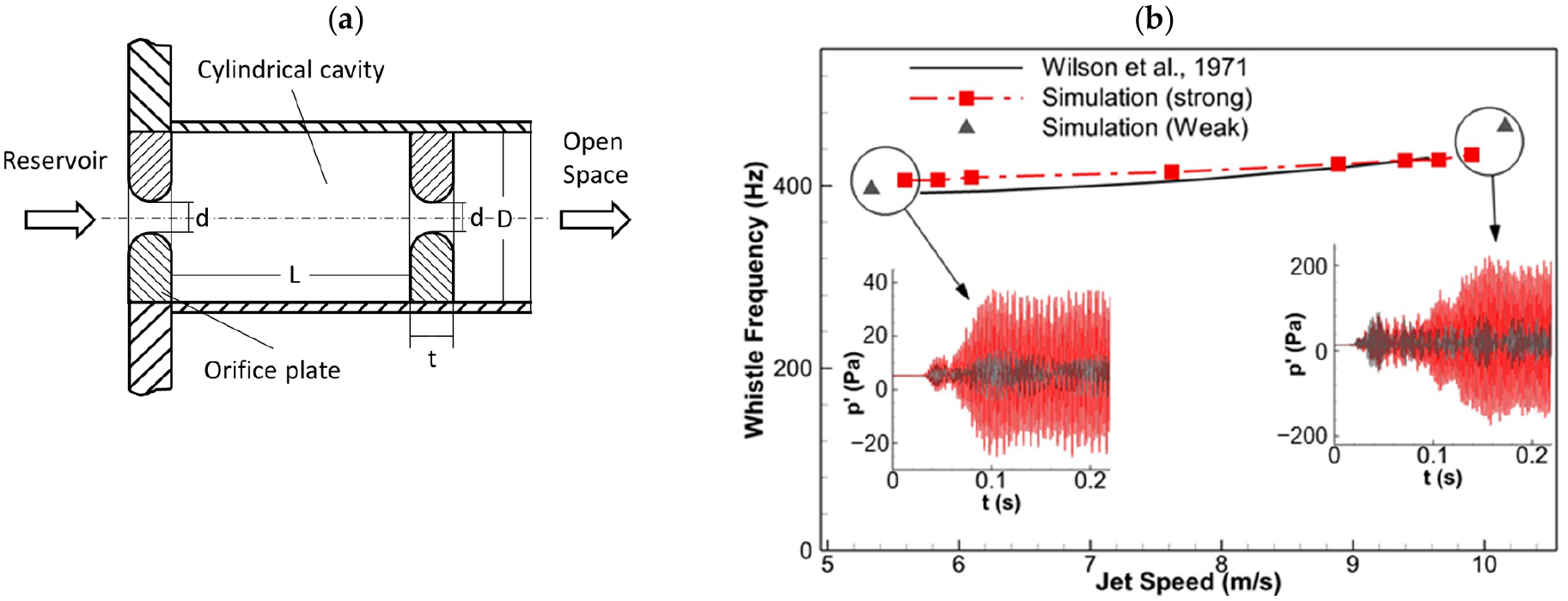
Validation case and result. (**a**) The whistle configuration used in [33]. The geometry selected for validation was, in centimeters, L = 5.08, d = 0.635, t = 1.27, and D = 5.18. (**b**) Comparison between the simulation-predicted and experiment-measured resonance frequencies against the jet speed for the selected whistle configuration. The subfigures compare amplitudes of pressure signals.

Since acoustic feedback and resonance were involved in the production of the whistling sound, it was critical that the simulation setup was able to resolve acoustic feedback and accurately predict resonance frequencies. To validate this, an experimental whistle configuration was chosen from reference [33], which examined the whistling frequency in a range of flow speeds and geometric parameters. The experiment was designed to investigate the human oral whistle, and it had comparable geometric dimensions and Reynolds numbers to the current issue of interest. The configuration was axisymmetric with a cylindrical cavity enclosed by two identical concentric orifice plates with rounded edges (Figure 3a). The upstream orifice was connected to a large reservoir, and the downstream orifice opened up to free space. Whistling occurred within a limited range of jet speeds when air was blown through the device, and the frequency of the whistling depended on the geometric parameters. A 1:1 geometry model was generated for numerical validation. The simulation setup (including the domain, grid resolution, and solver settings) was similar to that of the SGS simulations, except for the use of a large open tube (20D) upstream of the first orifice to mimic the experiment’s reservoir. A pressure probe located at the center of the cylindrical cavity near the wall was used to determine the resonance frequency, and the jet speed was varied to cover the range reported in [33], with the corresponding Reynolds numbers ranging from 2000 to 4200.

Figure 3b shows the comparison of the resonance frequencies between the simulations and experiments in a range of jet speeds for the selected whistling configuration. The results show a good overall match with errors below 4% for the resonance frequency. The simulation also predicts the trend of the resonance frequency increasing with jet speed. The range of jet speeds where whistling occurred in the original experiment was reported to be 225 to 380 in/s (5.72 to 9.65 m/s). In the simulations, when the jet speed was outside the resonance range (the grey triangles in Figure 3b), the pressure signal (the grey lines in the subfigures) became much weaker, indicating the offset of resonance. The estimated range of the resonance was 5.59 to 9.91 m/s. This validation case demonstrates that the current setup accurately predicts the resonance frequency and the jet speed range for whistling, indicating that the setup was capable of capturing both the acoustic feedback and flow-sustained tones.

### 2.4. Parametric Variation

To examine how glottal configuration affects the SGS sound, we considered two different glottal opening sizes, 1.1 cm and 0.4 cm in diameter, which we refer to as the neutral glottis and the constricted glottis, respectively. The sizes were calculated by converting typical glottal area data [50–52] to equivalent opening sizes in the axisymmetric configuration.

The parametric variables for each glottal configuration were the severity of the SGS, the location of the SGS, and the flowrate. The severity of the SGS was represented by the percentage of the area blockage of the trachea, and severity and blockage are used interchangeably in this paper. Five levels of severity were considered with area blockages of 51%, 75%, 84%, 91%, and 96%, corresponding to stenotic opening diameters of 1.4, 1.0, 0.8, 0.6, and 0.4 cm. Five flowrates (100, 300, 500, 700, and 1000 mL/s) were considered to cover a range of respiratory rates based on previous measurements [53] and similar studies [12,54]. Three locations of SGS were also considered with distances of 1.0, 2.0, and 3.0 cm. The Reynolds numbers of all the cases ranged from 750 to about 20,000 based on the average volumetric velocity and diameter at the smaller of the stenotic and glottal openings.

## 3. Results

The results are organized in three subsections. Cases with the neutral glottis are presented in Section 3.1. Cases with the constricted glottis are presented in Section 3.2. The effect of SGS distance is presented in Section 3.3. Throughout the presentation of the results, the term purity is used to compare the tonal sound of various cases. Qualitatively, a spectrum with a single-point spike is considered to be the purest. Here, we define the purity of the sound as the energy ratio of the 100 Hz band containing the highest peak in the spectra to the total energy from 0 to 6000 Hz. Specifically, it is calculated from the discrete Fourier transform of the acoustic signal as

$$\text{Purity}≔\sum_{j=j_{\text{peak}}-2}^{j_{\text{peak}}+2}Y_{j}^{2}/\sum_{i=1}^{300}Y_{i}^{2},$$

where *Y* is the amplitude of the spectrum and the subscript represents the index of discrete frequency. The index *j*_*peak*_ is the index of the highest spectral peak. The range of ±2 and 1–300 specifies the bandwidth for the tonal peak and the total energy since the resolution of the discrete Fourier transform is 20 Hz.

The overall sound pressure level (SPL) is calculated using the signal from the last 50 ms of the simulation as

$$\text{SPL}≔20{log}_{10}\frac{\text{RMS}(p(t))}{20\;\mu \text{Pa}},60\;\text{ms}\leq t\leq 110\;\text{ms},$$

where RMS is root mean square and *p* is acoustic pressure.

### 3.1. Neutral Glottis

#### 3.1.1. Sound Pressure Level under Neutral Glottis

**Figure 4.**
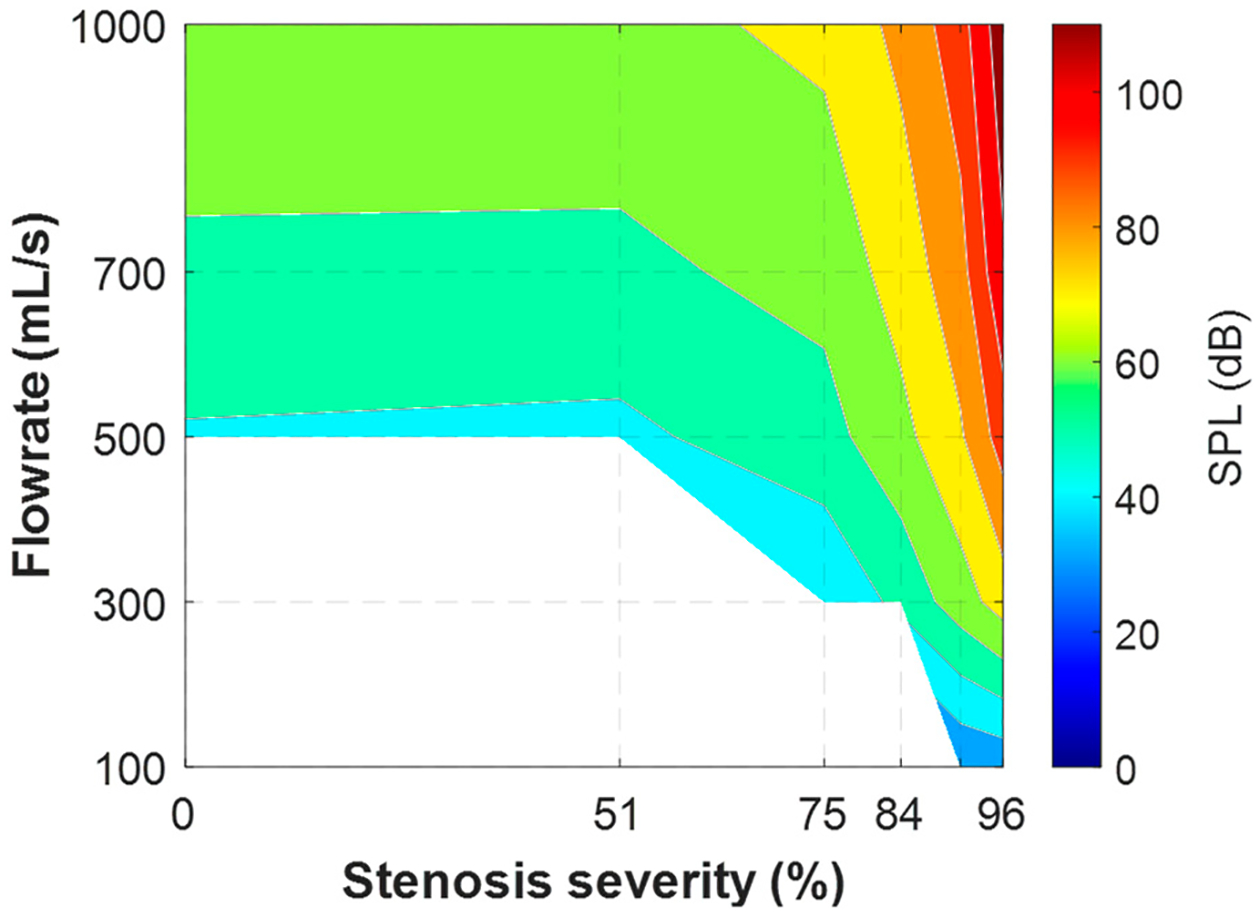
Overall sound pressure level of parametric cases for the neutral glottis and an SGS distance of 2.0 cm.

Figure 4 shows the overall sound pressure level for parametric cases with the neutral glottis (*g* = 1.1 cm) and a fixed SGS distance of 2.0 cm. The cases with an SPL below 0 dB are left blank, as they are below the threshold of human hearing. The results reveal that the sound is mostly inaudible when the flowrate is below 500 mL/s except for with high severity. Additionally, the impact of the SGS on the SPL is negligible when the severity is lower than 50%, and the SPL is mostly determined by the flowrate, reaching a maximum of approximately 60 dB at a flowrate of 1000 mL/s. As the severity increases beyond 50% to 75%, the SPL begins to increase, and as the severity surpasses 75%, the SPL sees a rapid increase and becomes more influenced by the severity of the SGS. At 96% obstruction, the sound is audible at almost all flowrates, and reaches almost 110 dB at 1000 mL/s.

#### 3.1.2. Sound Spectrum under Neutral Glottis

**Figure 5.**
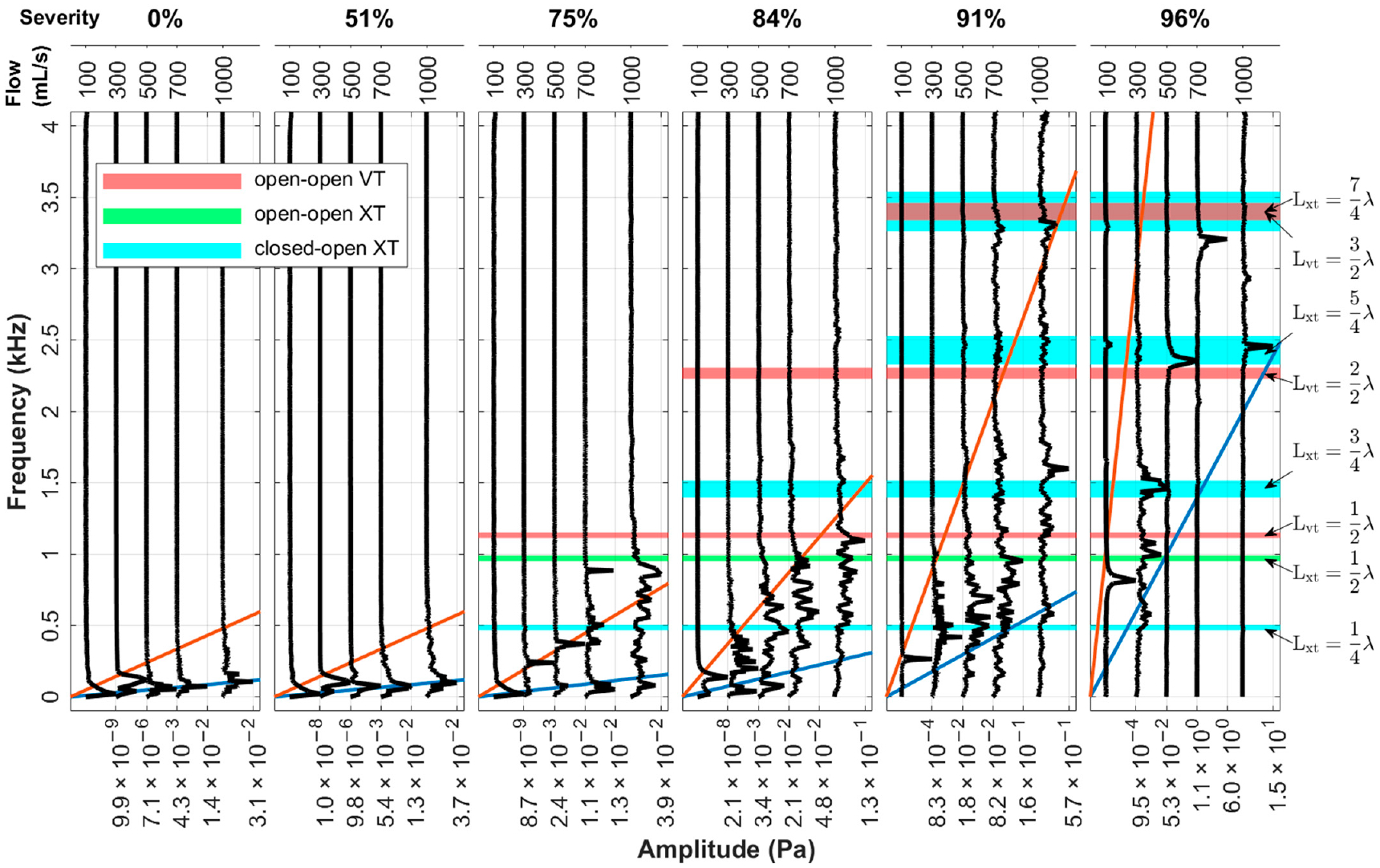
Spectra of acoustic pressure for cases with an SGS distance of 2.0 cm and the neutral glottis. The amplitude (acoustic pressure in Pa) is normalized to [0, 1] in the spectrum plot with maximum peak marked for each case at the bottom axis. The blue oblique line represents a constant St = 0.1 and the orange line St = 0.5, calculated for the glottal jet in the first two subfigures and the SGS jet in the rest.

Figure 5 depicts the spectra for the neutral glottis with a fixed SGS distance of 2.0 cm. The spectra are divided into subfigures, each containing five cases that share the same SGS severity but have increasing flowrates (shown at the top). Each black curve in the subfigures represents the spectrum of the acoustic pressure of a single case. The amplitude of the spectra is normalized with respect to the highest peak of each case. The spectrum is shown up to 4000 Hz, beyond which the amplitude is almost zero. To aid in the interpretation of the spectra, shaded bands are included to represent estimated frequencies of various sources of acoustic resonance, including the harmonics of the vocal tract (VT) from the glottis to the mouth and the harmonics of the extended tract (XT) from the SGS to the mouth (see Figure 1c). Note that, depending on the size of the SGS and the glottis, the vocal tract and extended tract can be considered as having an open–open (O-O) or a closed–open (X-O) end condition. The frequencies of the harmonics are estimated using the formulas for straight tubes with ideal O-O or X-O end conditions, and the specific acoustic modes are annotated to the right. The bands are thickened to suggest a larger error in the estimated frequencies for higher harmonics [55]. A detailed explanation of the frequency estimates is provided in Appendix A. The characterization of the tonal sound is based on the matching between the spectrum and these frequency source estimates. Two oblique lines in the subfigures indicate a constant Strouhal number (St) of 0.1 (blue line) and 0.5 (orange line), which are calculated based on the size of the smaller orifice in the system and its average jet velocity.

When there is no SGS present (a 0% blockage), the spectra show a hump around a low frequency that increases with flowrate. This frequency originates from the instability of the glottal jet, corresponding to a constant Strouhal number (St) of 0.1 (blue line). The tones are classified as the jet tone from the glottis. For the cases with a blockage of 51%, the effect of the SGS is almost negligible, and the spectra are similar to those of 0% blockage cases.

At an SGS blockage of 75%, the low frequency peak resulting from the glottal jet can also be observed for most cases, yet it is less dominant. A single narrow frequency peak appears at flowrates of 300 mL/s and 500 mL/s, which is found to match the vortex shedding frequency of the SGS jet, indicating that it is a hole tone of the SGS jet. As the flowrate increases, the first acoustic harmonic of the open–open XT begins to appear at 700 mL/s and becomes dominant at 1000 mL/s.

At an SGS blockage of 84%, most spectra become noisy with peaks of tapering amplitude around tract harmonics. At an SGS blockage of 91%, a pure SGS hole tone of 265 Hz is excited under a flowrate of 100 mL/s. As the flowrate increases to 300 mL/s and beyond, the spectra become cluttered with peaks around tract harmonics. The disappearance of the pure hole tones at 300 and 500 mL/s seen in 75% severity could be related to the relative size of the obstacle hole (the glottal opening) and the orifice (the stenotic opening). The diameter ratio of the hole to the orifice is 1.4 at 84% severity and 1.8 at 91% severity compared to 1.1 at 75% severity. Chanaud and Powell [14] demonstrated that when the hole diameter was increased to twice the size of the orifice, the sound pressure became barely measurable.

At an SGS blockage of 96%, a pure SGS hole tone of 816 Hz is excited at 100 mL/s. At 300 mL/s, the spectrum shows tonal peaks but is not pure. The major peak in the spectrum is the second harmonic of the closed–open XT. At 500 mL/s, a pure tone of 2357 Hz is excited, which is close to the 3rd harmonic of the closed–open XT. The spatial waveform shows a wavelength that is slightly less than *L*_vt_. The pressure node is downstream of the glottis exit, which indicates that the open–open vocal tract mode is not excited. At 700 mL/s, a strong pure tone is excited at 3286 Hz, which is very close to the overlapping frequencies of the 4th harmonic of the closed–open XT and the 3rd harmonic of the open–open VT. In this case, vortices are shed at the exit of the stenosis, and the wall pressure shows a 3/2 wavelength standing wave in the vocal tract (shown later in Figure 10a), corresponding to an open–open acoustic mode and a wavelength of 10 cm. The cavity length is roughly 2.5 cm, which is a 1/4 wavelength. Thus, a 7/4 wavelength standing wave is formed in the extended tract from the SGS to the mouth, with the glottal exit (x = 0 in Figure 1b) as a pressure node. This suggests that the acoustic modes of both the open–open VT and the closed–open XT are active in this case, which is possibly due to the specific vocal tract length and the SGS distance. At 1000 mL/s, a strong pure tone of 2455.5 Hz is excited, corresponding to the third harmonic of the XT with the closed–open end condition. The acoustic mode is a 5/4 wavelength standing wave, similar to the 500 mL/s case. The excited mode at 1000 mL/s is lower than that at 700 mL/s. This is probably due to the increase in the vortex convection speed with flowrate causing the coupling between the hole tone and the tract resonance to settle at a lower vortex mode. Inspection of the flow field confirms this as the number of vortices between the SGS and the glottis changes from two to one. The readers are referred to references [34,35] for a more detailed and illustrated presentation of the nonlinear coupling phenomenon between the vortex mode of the jet–obstacle system and the acoustic mode of the resonator. Briefly, the vortex shedding frequency (*f*_v_) of the jet scales with the convection speed and vortex mode in the form *f*_v_ ≈ *N*_v_*v*_C_/*L*_v_, where *N*_v_ is the number of vortices between orifice and obstacle, *v*_C_ is the vortex convection speed, and *L*_v_ is the vortex travelling distance. Lock-in occurs when the shedding frequency is in the proximity of an acoustic mode of the resonator represented by $f_{\text{r}}\in \left\{f_{\text{r}}^{1},f_{\text{r}}^{2},f_{\text{r}}^{3},\cdots ,f_{\text{r}}^{n}\right\}$, where the superscript numeral represents the order of the acoustic modes. As the convection speed increases (roughly linearly) with the jet speed, the shedding frequency tends to increase and eventually deviates too much from the acoustic mode to stay locked-in. Before it reaches the next higher acoustic mode, it is possible for the vortex shedding to switch to a lower mode to lock in to a lower acoustic mode if a solution exists for the coupling *f*_v_ ≈ *f*_r_ at an intermediate flow speed.

### 3.2. Constricted Glottis

#### 3.2.1. Sound Pressure Level under Constricted Glottis

**Figure 6.**
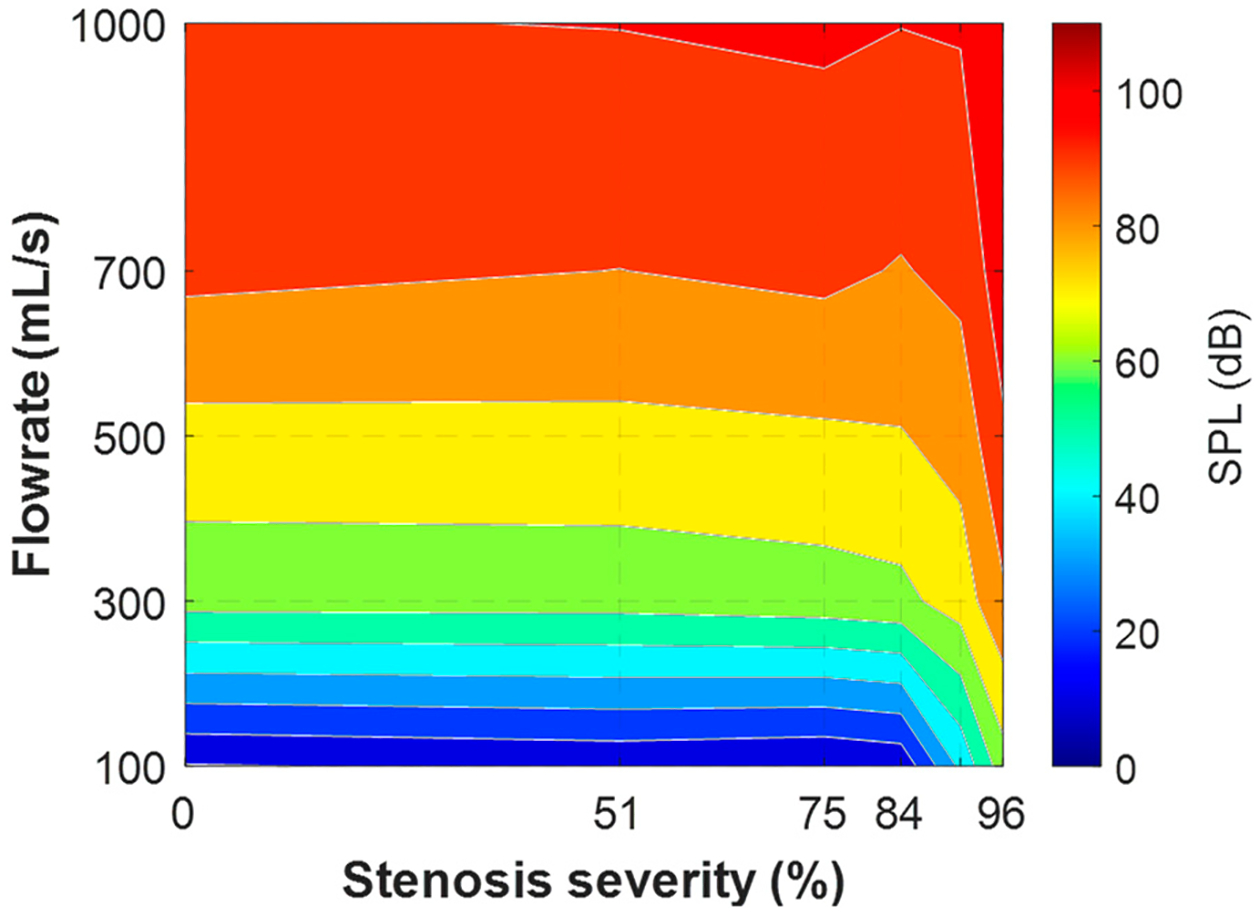
Overall sound pressure level of parametric cases for the constricted glottis and an SGS distance of 2.0 cm.

Figure 6 presents the overall sound pressure level (SPL) for parametric cases with the constricted glottis. Unlike the neutral glottis cases, the SPL is mostly determined by the flowrate alone, except for with the highest SGS severity. Even without any blockages due to SGS, the SPL can almost reach the highest level because the constricted glottis acts as a blockage itself. Since the glottal blockage is the same as in the most severe SGS blockage (96%), the SPL is almost not affected by the severity of the SGS until it reaches the same level of blockage. The maximum SPL is comparable to that in the neutral glottis cases.

#### 3.2.2. Sound Spectrum under Constricted Glottis

**Figure 7.**
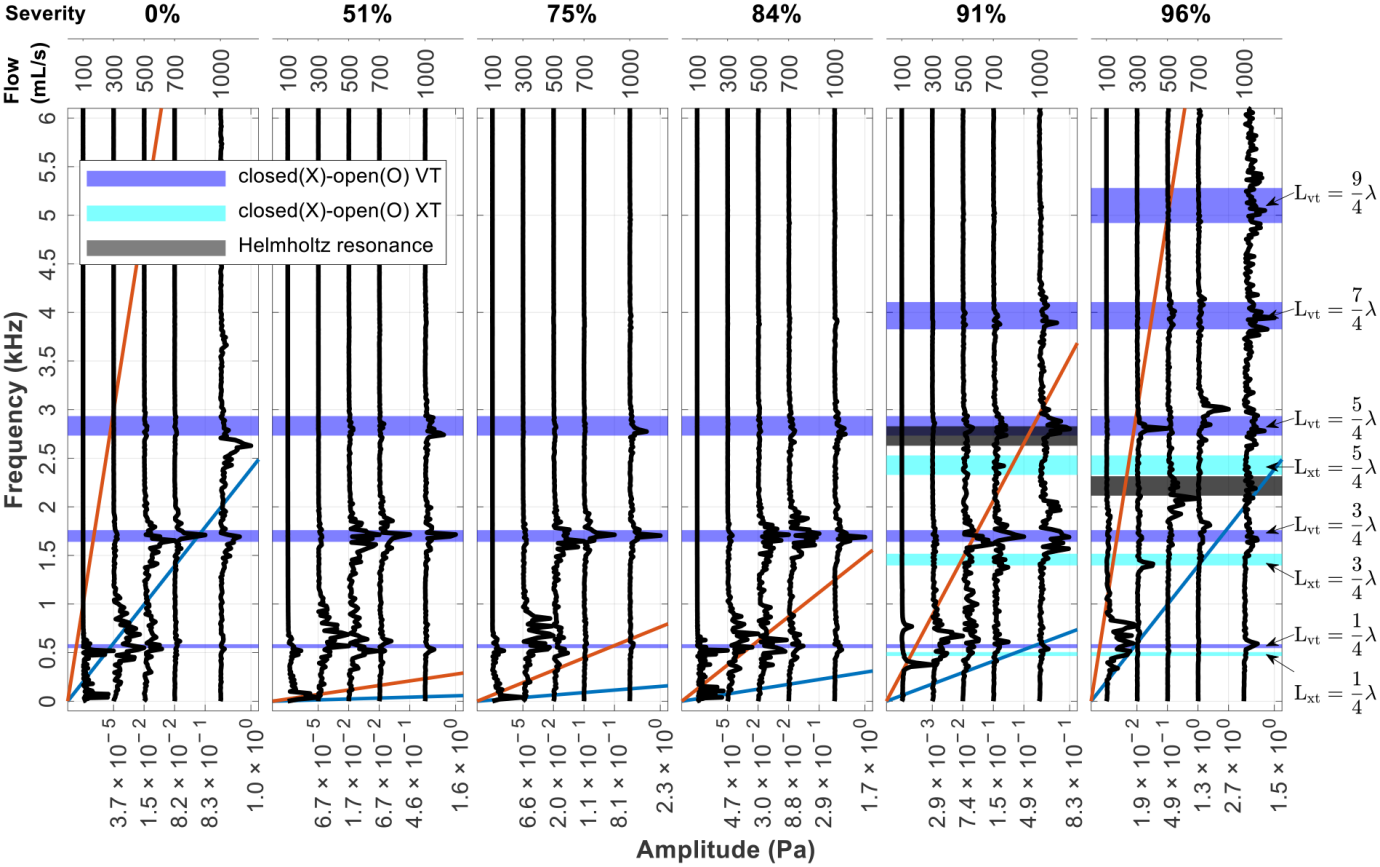
Spectra of acoustic pressure for cases with the constricted glottis and an SGS distance of 2.0 cm. The layout is the same as Figure 5. The constant St lines are based on the SGS except in the first subfigure, where they are based on the glottis. The spectrum is shown up to 6000 Hz, beyond which the amplitude is almost zero.

Figure 7 displays the spectra for the constricted glottis configuration (*g* = 0.4 cm) at a fixed SGS distance of 2.0 cm using the same layout as Figure 5. The oblique lines indicating constant St are for the SGS jet except in the first subfigure, where they are for the glottal jet. The first observation is that it is mostly the harmonics of the X-O VT that are excited. In the absence of the SGS, the sound comes from the coupling between the vocal tract and the glottal jet (i.e., the pipe tone system). Higher harmonics become more dominant as flowrate increases. The purity of the tones varies with the flowrate. In most cases, clusters of peaks are observed around vocal tract harmonics. At 700 mL/s, a pure tone (1709 Hz) is excited, corresponding to the 2nd harmonic of the X-O VT. The spatial distribution of the wall pressure shows a 3/4 wavelength standing wave.

The effect of the SGS on the spectrum appears to be subtle but consistent. While the excited sound are still harmonics of the X-O VT, the dominant harmonics tend to shift toward the preferred St of the SGS jet and the hole tone system. Taking the cases with a 1000 mL/s flowrate as an example, the most dominant harmonic is the third one without SGS. As the severity increases, the second harmonic becomes dominant due to the lower frequency preferred by the SGS jet. This continues up to a severity of 84%. As the severity further increases to 91% and the preferred SGS jet frequency becomes higher, the third harmonic becomes dominant again and the fourth harmonic starts to appear on the spectrum. At 96% severity, the fourth harmonic becomes dominant and even higher harmonics appear on the spectrum. The SGS also affects the purity of the excited tones. Taking 1000 mL/s cases again as an example, for a severity from 51% to 84% the spectra contain a single narrow dominant peak at the second harmonic of the X-O VT. In these cases, the shedding frequency of the SGS jet is locked to the VT harmonics, indicating the hole tone system and the pipe system are coupled into resonance. Indeed, the amplitude of the peaks are slightly higher than when SGS is absent. For severities of 91% and 96%, the peaks are more distributed, and no resonance is formed. In the 91% severity case, a hump around 2000 Hz can be seen, which is from the hole tone system. The same influence of the SGS can be observed for cases with a different flowrate.

Secondly, a few special cases are noted. For 91% severity at 100 mL/s, the hole tone mechanism is dominant. For 96% severity at 300 mL/s, a pure tone is excited at the frequency of the third harmonic of the X-O VT (2806 Hz) with a weak subharmonic of about half the frequency (1413 Hz). Inspection of the flow field animation confirms that the frequency of this subharmonic is the same as the vortex shedding frequency of the SGS jet. For 96% severity at 500 mL/s, the peak of the spectrum aligns with none of the X-O tract harmonics. Instead, the frequency is close to the estimated resonance frequency of the Helmholtz resonator formed by the SGS, the glottis, and the cavity in-between (Figure 1c; see Appendix A for the estimation of the Helmholtz resonance frequency). Inspection of the acoustic field shows that the acoustic pressure on the cavity wall oscillates uniformly, and the glottal exit is close to a pressure node.

Lastly, the spectra show that harmonics of the extended tract are not excited due to it being broken by the constricted glottis.

#### 3.2.3. Comparison with Neutral Glottis

**Figure 8.**
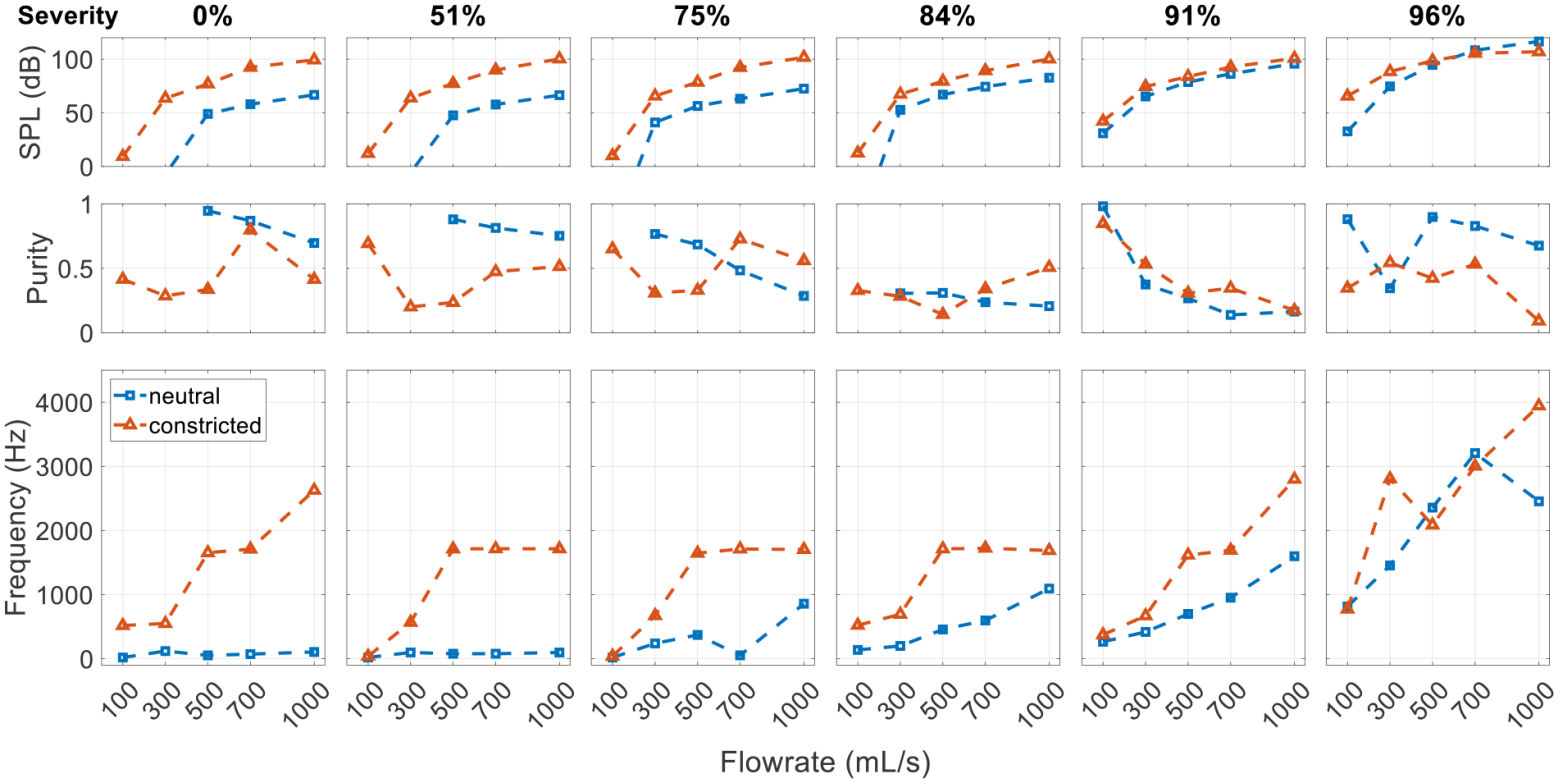
Effect of glottal configuration on the acoustic spectrum. Comparison is made between cases with the neutral and constricted glottis and an SGS distance of 2.0 cm.

It can be seen from the spectra that constricting the glottis has a significant influence on the sound. To facilitate comparison, Figure 8 presents the SPL, purity, and the dominant frequency (corresponding to the highest peak in the spectra) for the corresponding cases with different glottal configurations. With the constricted glottis, the pitch and SPL are generally higher except for in the most severe SGS cases. At a low SGS severity (<50%), the weak glottal jet tones in the neutral glottis cases are replaced by strong tract harmonics. At a medium SGS severity (75%), the SGS hole tones are also replaced by the tract harmonics. At the highest severity (96%), the excited tones in both configurations are tract harmonics-dominant, but the effective length of the resonant tract is slightly different. The effective tract length for the neutral glottis configuration is slightly longer with the SGS functioning as the closed end. However, the change of the dominant frequency is difficult to predict as it depends on the acoustic mode and changes nonlinearly with the flowrate. The purity of the tones is lower at this severity.

### 3.3. Effect of SGS Distance

**Figure 9.**
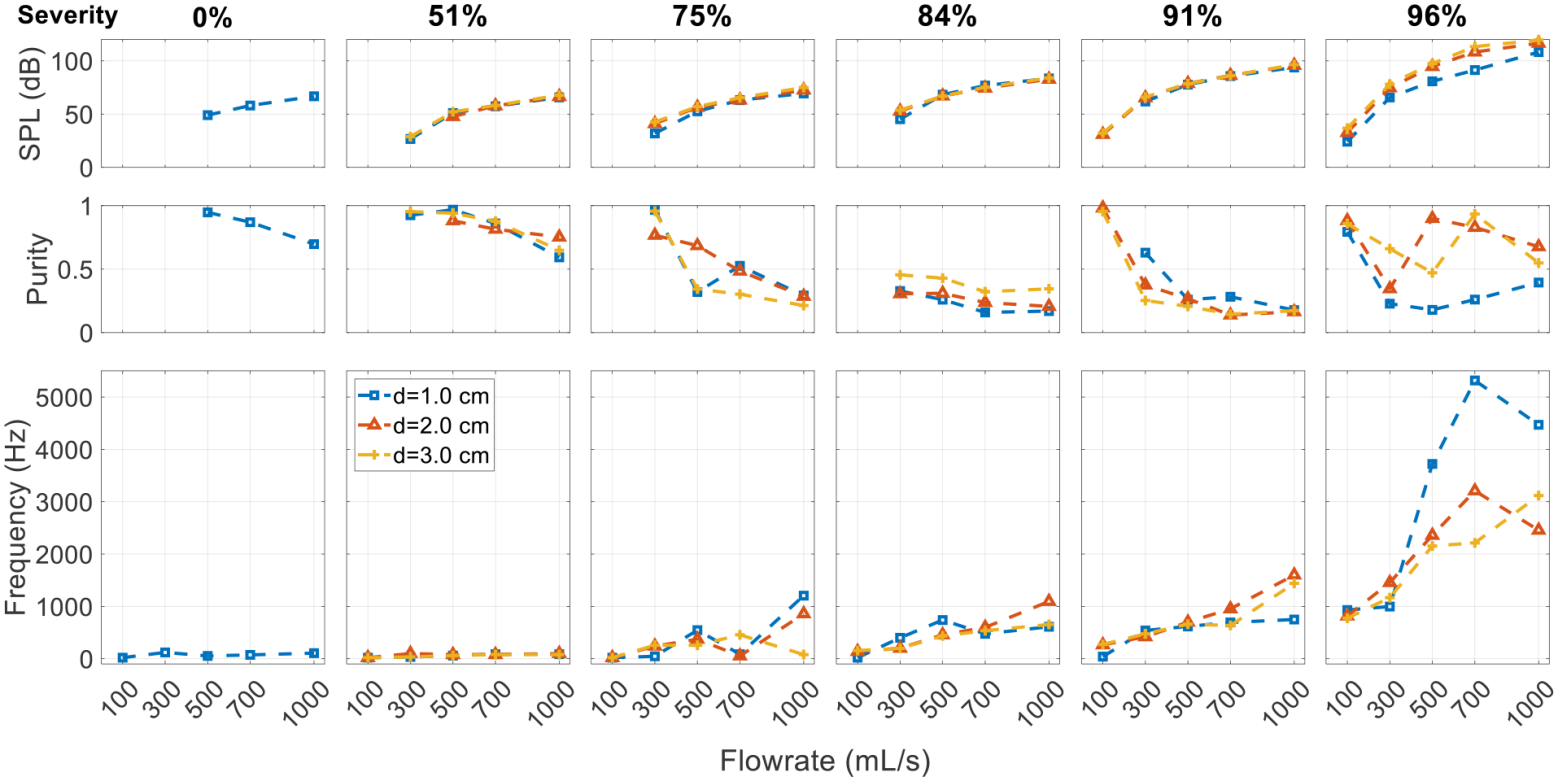
Effect of stenosis distance on the expiratory sound. Comparison is made among cases with the neutral glottis but different SGS distances.

When altering the distance of the SGS, similar spectra patterns and SPL to those shown in the previous two sections are observed. For the sake of brevity, the SPL and the spectra of these cases are not displayed. Instead, comparisons are made in a similar way to Figure 8, but for different SGS distances. The location of the SGS affects the length of the extended tract, as well as the travel distance of the SGS jet before impinging on the vocal folds. As a result, it also affects the coupling between the hole tone system and the tract. In Figure 9, the effect of the SGS distance in the neutral glottis cases is demonstrated. Compared to the SGS severity and flowrate, the effect of stenosis distance on the overall SPL is negligible. A slightly higher SPL can be observed when the stenosis is farther from the glottis, but the overall difference is very subtle. The influence of the SGS distance on tonal purity is complex. For 96% severity, the *d* = 1.0 cm cases tend to have significantly lower purity.

**Figure 10.**
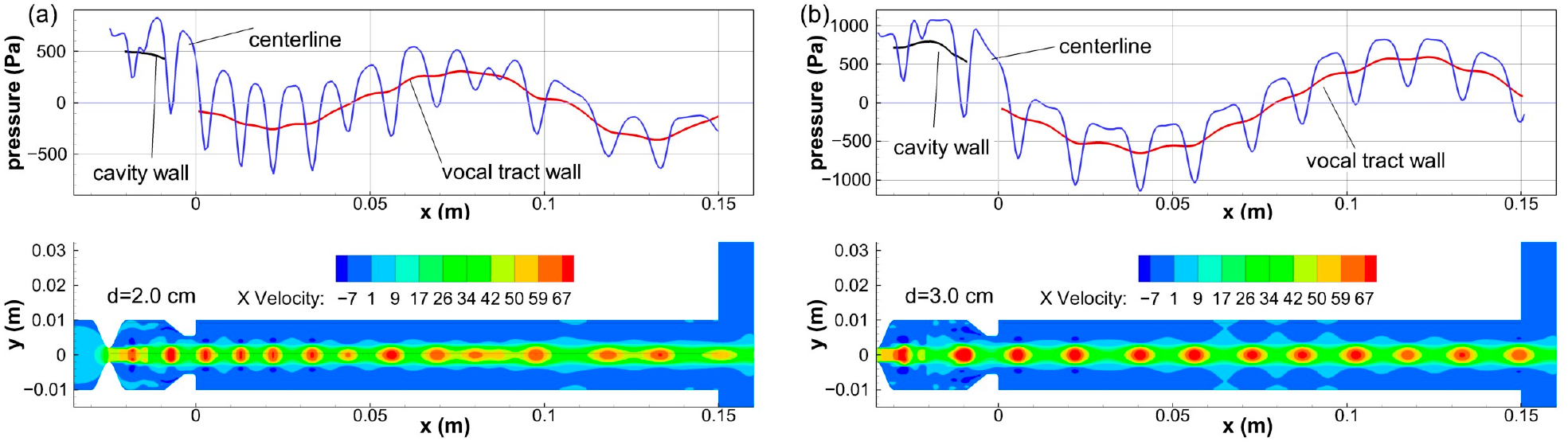
Acoustic mode change due to stenosis distance. (**a**) *d* = 2.0 cm. 3/2 wave in vocal tract and quarter wave in SGS cavity. (**b**) *d* = 3.0 cm. Single wave in vocal tract and quarter wave in SGS cavity.

At the highest severity of SGS, tones of lower pitches are generally excited as the stenosis moves away from the glottis with a few exceptions. This is due to two contributing factors: (1) the length of the extended vocal tract increases, leading to decreases in the frequencies of tract harmonics, (2) the longer travel distance of the stenosis jet tends to reduce the hole tone frequency, which is particularly evident in the 700 mL/s cases. As the stenosis distance increases from 1.0 cm to 2.0 cm to 3.0 cm, the sound frequency decreases from about 5400 Hz to about 3300 Hz and then to about 2300 Hz. The pure excitation in these cases allows for a clear view of a standing wave in the vocal tract. Figure 10 illustrates the acoustic mode of the *d* = 2.0 cm case and the *d* = 3.0 cm case. Since the pressure drop along the tract is low, the acoustic pressure can be approximated by the gauge pressure near the wall. The centerline pressure is the superposition of the acoustic pressure and the flow pressure. Compared to the quarter wave plus a 3/2 wave mode observed in the *d* = 2.0 cm case, the *d* = 3.0 cm case has a single wave in the vocal tract, which is the next lower acoustic mode. In both cases, the glottal exit is a pressure node.

For cases with pure excitations, orderly shedding of vortices can also be observed. The contours in the bottom row of Figure 10 show the spatial oscillation of the axial component of the velocity (the x velocity), which arises due to the coupling of the stenosis jet and the acoustic feedback near the exit of the SGS. Both cases have two vortices between the SGS and the vocal folds. However, the *d* = 3.0 cm case has a lower vortex shedding frequency due to the longer distance.

**Figure 11.**
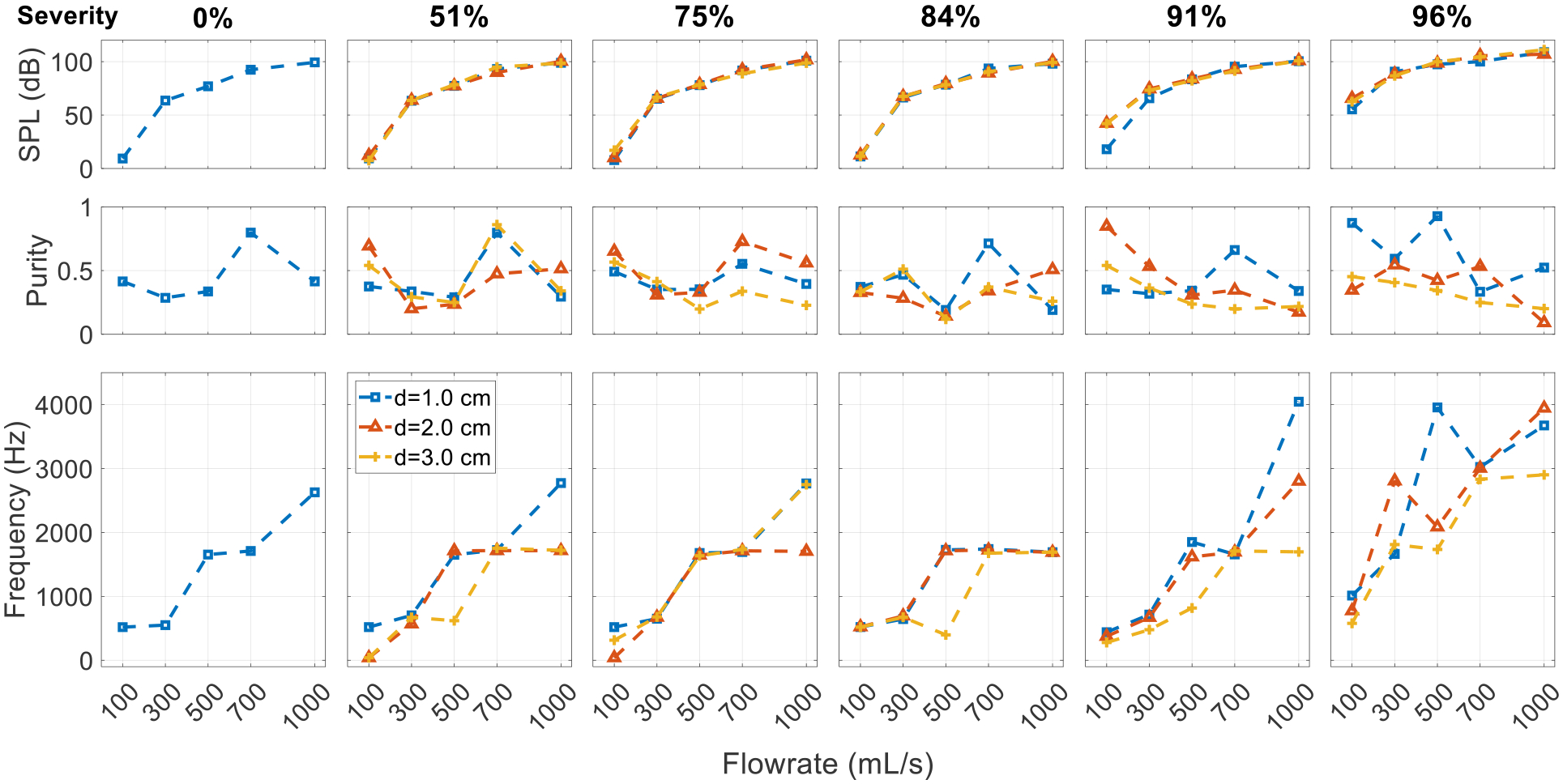
Effect of stenosis distance on the expiratory sound. Comparison is made among cases with the constricted glottis but different SGS distances.

Figure 11 shows the effect of the SGS distance in the constricted glottis cases. Like in the neutral glottis cases, the SGS distance has a negligible effect on the overall SPL, and its influence on purity is complex. In general, the most dominant tract harmonic is not affected by the stenosis distance until the severity is very high. Similar to the neutral glottis cases, the pitch of the sound tends to be higher when the SGS is closer to the vocal tract.

## 4. Summary and Discussion

### 4.1. Tonal Sound Mechanisms

For airways with SGS, the primary source of tonal sound generation during expiration was the jet–obstacle system in which the SGS produced the jet and the vocal folds function as the obstacle. Additionally, a few resonators were identified that coupled with the jet–obstacle system to form a strong excitation of pure tones, including the vocal tract (from glottis to mouth) and the extended tract (from the SGS to the mouth). The purity and the frequency of the tones depended on the coupling between the jet–obstacle system and the resonator, which was affected by the flowrate in a nonlinear fashion due to possible changes of acoustic mode and vortex mode.

Pure but weak hole tones were observed, starting from a medium SGS severity (>50%) at low flowrates. The frequency of the tones was not affected by acoustic feedback from the tract, which was also weak due to the low sound level. The frequency of the hole tones tended to be relatively low.

As the flowrate and the sound pressure level increased, the acoustic feedback of the resonators had stronger influence on the SGS jet. However, resonance only occurred in cases with 96% severity, in which the frequency of the SGS jet locked to the excited harmonic of the resonator. For medium to high severity (84% to 91%), while the harmonics of the resonator were present, the spectra were cluttered with various peaks. This could be due to two factors: (1) the acoustic feedback from the tract was still not strong enough to lock the hole tone system to the same frequency, (2) a compatible coupling could not be established under the specific geometric configuration.

A particular interesting configuration was noticed where the closed–open extended tract and the open–open vocal tract had overlapping harmonics. A pure tone could be excited at the overlapping frequency under a flowrate favorable to the hole tone system. In this case, the distance of the SGS could be inferred from the frequency.

When constricting the glottis to a very small opening size, the harmonics of the closed–open vocal tract became dominant, resulting in a higher overall SPL and pitch. Again, the purity of the tones depended on the coupling between the hole tone system and the vocal tract. At high severity, the cavity between the SGS and the vocal folds could form a Helmholtz resonator, which, on very rare occasions, could lock the excited tone to the Helmholtz resonance frequency.

### 4.2. Clinical Implications

The current simulation results indicate that strong tonal excitation is only observed under a high SGS severity. Since high severity can cause severe consequences, urgent visual inspection that can detect the SGS directly may be needed. This may limit the usefulness of sound signals as a diagnostic tool. However, misdiagnosis has been reported even in the most severe conditions. For instance, Spittle & McCluskey [9] reported a case where a patient with exertional dyspnea and wheeze was treated as acute asthma. The patient’s condition deteriorated despite the treatment. It was not until a failure to insert a ventilation tube into the patient’s trachea that the authors found the severe SGS in the patient. In hindsight, the patient presented a virtually silent chest, suggesting that the source of the wheeze was in the upper airway instead of the bronchi. In such cases, it may be possible to make the correct diagnosis at an earlier stage if the acoustic information is considered.

The tonal characteristics of the SGS sound are highly variable but when combined with factors such as the respiration phase, volume, duration, rate of onset, and associated symptoms, the tonal characteristics of the sound can serve as an indicator of the location and size of the airway obstruction [2]. This current study suggests that developing different glottal tasks can provide more acoustic information for differential diagnosis, which is encouraging since the SGS sound is more affected by glottal configuration. Basovsky et al. [13] hypothesized and then experimentally showed that tonal sound in the bronchi is generated by nozzle–jet–obstacle structures formed in obstructed bronchial airways, which are decoupled from upper airways. Thus, we expect that respiratory sounds from lower airway sources are less influenced by glottal and vocal tract configuration. Additionally, since the tonal components predominantly come from tract harmonics, changes in vocal tract shape and length that alter the resonance frequency are expected to affect the tonal characteristics of the SGS sound. Here we suggest a simple hypothetical test for differentiating subglottic obstruction from bronchial obstruction: patients who present with tonal respiratory sounds can be asked to produce the /h/ sound during either inhalation or exhalation, which essentially modifies the glottis from being neutral to constricted. If the tonal sound changes or disappears during this operation, then the sound is likely to be from a subglottic obstruction. However, further clinical verification and testing are required to develop corresponding methods for these tasks. Cross validation between the acoustic approach and other approaches such as electromyography of the respiratory muscles (see, e.g., [56]) may lead to the development of more robust and reliable non-invasive procedures to diagnose airway obstruction.

It is worth noting that the current simplified configuration is more likely to have orderly flow structures and tonal sound, whereas realistic cases are expected to have a much more complex acoustic spectrum.

### 4.3. Limitations

To properly interpret the results of the current simulation, it is crucial to acknowledge the simplifications made in the setup and how they diverge from real-world scenarios. The most significant simplification is the assumption of axisymmetric geometry, which eliminates turbulence from the flow and promotes more ordered flow patterns. In reality, the glottis takes on a triangular shape and generates a two-dimensional jet, where the bimodal lateral jet instability could play a more significant role in the sound production [57]. This may lead to different tonal mechanisms or even completely turbulent sounds. Under such conditions, the pipe tone from the glottal jet may be weak or entirely absent. It is also more difficult to form a Helmholtz resonator by constricting the glottis, because the glottal opening becomes a slit instead of a hole.

In addition to the pipe tone, the hole tone is another important mechanism contributing to the tonal sound of the SGS. Understanding the effects of axisymmetry on the hole tone is also crucial. The work of Chanaud and Powell [14] revealed that the jet disturbance remained symmetric even when the downstream obstacle was a long rectangular slit rather than a hole. Moreover, jet flows were found to be predominantly pressure-sensitive, regardless of the direction of the external source [14,27,29]. It is therefore reasonable to expect the hole tone to be present in a realistic, non-axisymmetric glottal configuration. However, the tones are expected to be less pure compared to the spectra obtained using the axisymmetric configuration, due to the complex flow patterns associated with non-axisymmetric geometries.

Although the SGS is commonly modeled using simplified geometries such as axisymmetric [17,18]) or two-dimensional [19] shapes, irregular shapes of the SGS are commonly observed in clinical examination. Compared to the axisymmetric SGS, the irregularly shaped SGS is less likely to produce whistling tones, likely due to the irregularities in the shape that disrupt the orderly flow structures necessary for whistling to occur. Furthermore, all tissues are modeled as rigid walls without considering possible vibratory and damping effects that could potentially absorb energy from the airflow and acoustic feedback and weaken the tonal sound.

This current study focuses solely on expiration, while reports suggest that SGS stridor is often inspiratory or biphasic [11]. Based on these current findings, it is expected that the inspiratory tonal sound in the SGS will also be characterized by pipe tones that are independent of the flow direction [39]. However, the tonal quality during inspiration may differ as the hole tone mechanism is absent. Future studies are needed to confirm this. Additionally, investigating how glottal configuration affects inspiratory sound could aid in distinguishing SGS from other respiratory disorders that produce a similar sound.

## Figures and Tables

**Table 1.** Simulation setup summary.

Solver options	Solver type	Spatial discretization		Time integration
		Pressure	Others	
	Pressure based	2nd order	QUICK	2nd order implicit
Air properties	Viscosity	Thermal conductivity	Specific heat	Molecular weight
	1.7894 × 10^−5^ kg/(m s)	0.0242 W/(m K)	1006.43 J/(kg K)	28.966 kg/kmol
Domain parameters	Trachea length	Vocal tract length	Tract diameter	Environmental space radius
	10.0 cm	15.0 cm	2.0 cm	2.0 m

**Table 2.** Solution comparison between the two densest meshes.

Flowrate	Metrics	73 K Cells	105 K Cells	Error
300 mL/s	Overall SPL(dB)	86.1	89.6	3.5 (dB)
	Primary frequency (Hz)	1785	1806	1.16%
1000 mL/s	Overall SPL (dB)	111.0	113.1	2.1 (dB)
	Primary frequency (Hz)	2897	2941	1.50%

## Data Availability

The data that support the findings of this study are available from the corresponding author upon reasonable request. The data are not publicly available due to data volume and complexity.

## Appendix A. Estimation of Frequencies of Resonant Sources

The resonant frequencies of the vocal tract and the extended tract are estimated based on standing waves inside a pipe with ideal closed–open or open–open end conditions. For a closed–open tract, the frequency of the *n*th harmonic is

$$f_{n}=\frac{(2n-1)c}{4L}\pm n\Delta \text{F},n=1,2,3,\ldots$$

where *c* is the speed of sound, and *L* is the length of the tract. The ±*n*ΔF part is used to take into account possible errors in the estimation. ΔF = 20 Hz is used, which is the resolution of the Fourier transform in this study. Similarly, for an open–open tract, the frequency of the *n*th harmonic is

$$f_{n}=\frac{nc}{2L}\pm n\Delta \;\text{F},n=1,2,3,\ldots$$

The resonant frequency of the axisymmetric jet-driven Helmholtz resonator is estimated using

$$f=\frac{c}{2\pi }\sqrt{\frac{1}{V}\left(\frac{A_{1}}{L_{e1}}+\frac{A_{2}}{L_{e2}}\right)}\pm 100\;\text{Hz},$$

where *V* is the volume of the cavity, A is the area of a hole to the cavity, and *L*_*e*_ is the effective length of the holes on the surface bounding the cavity. The subscripts 1 and 2 refer to the upstream and downstream holes, respectively. This formula is similar to the formula for the typical Helmholtz resonator with one hole and can be obtained through some simple derivation assuming the mass in both holes vibrate in phase. In this study, the upstream hole is the SGS’s narrowing and the downstream hole is the glottis. The effective length is typically estimated as *L*_*e*_ = *L* + 0.3*D*, where *L* is the actual length of the hole and *D* is the diameter of the hold. In this study, the length of the glottal hole is 4 mm and the length of the SGS hole is estimated to be 1 mm.
